# Factors associated with suicide risk among Chinese adults: A prospective cohort study of 0.5 million individuals

**DOI:** 10.1371/journal.pmed.1003545

**Published:** 2021-03-11

**Authors:** Rongqin Yu, Yiping Chen, Liming Li, Junshi Chen, Yu Guo, Zheng Bian, Jun Lv, Canqing Yu, Xianmin Xie, Dan Huang, Zhengming Chen, Seena Fazel

**Affiliations:** 1 Department of Psychiatry, University of Oxford, Oxford, United Kingdom; 2 Medical Research Council Population Health Research Unit (PHRU), Nuffield Department of Population Health, University of Oxford, Oxford, United Kingdom; 3 Clinical Trial Service Unit & Epidemiological Studies Unit (CTSU), Nuffield Department of Population Health, University of Oxford, Oxford, United Kingdom; 4 Department of Epidemiology and Biostatistics, School of Public Health, Peking University Health Science Center, Beijing, China; 5 China National Centre for Food Safety Risk Assessment, Beijing, China; 6 Chinese Academy of Medical Sciences, Beijing, China; 7 Public Health Department, Pengzhou People’s Hospital, Chengdu, China; 8 Record Department, Pengzhou Traditional Chinese Medical Hospital, Chengdu, China; Massachusetts General Hospital, UNITED STATES

## Abstract

**Background:**

Suicide is a leading cause of death in China and accounts for about one-sixth of all suicides worldwide. The objective of this study was to examine the recent distribution of suicide and risk factors for death by suicide. Identifying underlying risk factors could benefit development of evidence-based prevention and intervention programs.

**Methods and findings:**

We conducted a prospective study, the China Kadoorie Biobank, of 512,715 individuals (41% men, mean age 52 years) from 10 (5 urban, 5 rural) areas which are diverse across China in geographic locations, social economic developmental stages, and prevalence of disease patterns. After the baseline measurements of risk factors during 2004 to 2008, participants were followed up for suicide outcomes including suicide and possible suicide deaths. Risk factors, such as sociodemographic factors and physical and mental health status, were assessed by semistructured interviews and self-report questionnaires. Suicide and possible suicide deaths were identified through linkage to the local death registries using ICD-10 codes. We conducted Cox regression to calculate hazard ratios (HRs) for suicide and for possible suicide in sensitivity analyses.

During an average follow-up period of 9.9 years, 520 (101 per 100,000) people died from suicide (51.3% male), and 79.8% of them lived in rural areas. Sociodemographic factors associated with increased suicide risk were male gender (adjusted hazard ratios [aHR] = 1.6 [95% CI 1.4 to 2.0], *p* < 0.001), older age (1.3 [1.2 to 1.5] by each 10-yr increase, *p* < 0.001), rural residence (2.6 [2.1 to 3.3], *p* < 0.001), and single status (1.7 [1.4 to 2.2], *p* < 0.001). Increased hazards were found for family-related stressful life events (aHR = 1.8 [1.2 to 1.9], *p* < 0.001) and for major physical illnesses (1.5 [1.3 to 1.9], *p* < 0.001). There were strong associations of suicide with a history of lifetime mental disorders (aHR = 9.6 [5.9 to 15.6], *p* < 0.001) and lifetime schizophrenia-spectrum disorders (11.0 [7.1 to 17.0], *p* < 0.001). Links between suicide risk and depressive disorders (aHR = 2.6 [1.4 to 4.8], *p* = 0.002) and generalized anxiety disorders (2.6 [1.0 to 7.1], *p* = 0.056) in the last 12 months, and sleep disorders (1.4 [1.2 to 1.7], *p* < 0.001) in the past month were also found. All HRs were adjusted for sociodemographic factors including gender, age, residence, single status, education, and income. The associations with possible suicide deaths were mostly similar to those with suicide deaths, although there was no clear link between possible suicide deaths and psychiatric factors such as depression and generalized anxiety disorders. A limitation of the study is that there is likely underreporting of mental disorders due to the use of self-report information for some diagnostic categories.

**Conclusions:**

In this study, we observed that a range of sociodemographic, lifestyle, stressful life events, physical, and mental health factors were associated with suicide in China. High-risk groups identified were elderly men in rural settings and individuals with mental disorders. These findings could form the basis of targeted approaches to reduce suicide mortality in China.

## Abstract 2

In a prospective cohort study, Rongqin Yu, Yiping Chen and colleagues investigate factors related to death by suicide among Chinese adults.

## Author summary

**Why was this study done?:**

- Suicide is a global health problem. However, there is a lack of research on risk factors in low- and medium-income countries, which account for around 80% of suicides worldwide.
- In China, there is limited evidence on modifiable risk factors for suicide, particularly from prospective studies with reliable measures of exposures.

**What did the researchers do and find?:**

- We conducted a prospective cohort study of half a million individuals from different regions in China. Semistructured interviews and self-reported questionnaires were administered to obtain data on sociodemographic, lifestyle, physical, and mental health factors, and individuals were followed for up to 10 years.
- We found strong associations with suicide deaths for stressful life events and specific mental disorders, including diagnoses of depression, schizophrenia-spectrum disorders, and sleep disorders.
- Furthermore, we identified elderly men in rural settings as a high-risk group.

**What do these findings mean?:**

- These findings suggest that addressing modifiable risk factors, such as by targeting high-risk groups like elderly men in rural areas, and improving public mental health services could be considered as part of any national suicide prevention strategy in China.

## Introduction

In China, around 130 000 people died from suicide in 2016, accounting for approximately one-sixth of suicides worldwide, at a rate similar to the global average [1]. Previous studies in China have reported major differences in the distribution of suicides from that in high-income countries [2,3], including higher rates in women and rural areas [4–6]. More recent investigations, however, suggest an epidemiological transition to a distribution seen in many high-income countries including higher risk in men [7], but these reports have been limited by small sample size and selected samples.

Although the effect of some risk factors has remained constant over time [2,3,7], this research is uncertain on the magnitude of effects and the effect of a range of modifiable risk factors, such as psychiatric disorders. Furthermore, most studies on suicide risk factors in China have been limited by cross-sectional and case-control designs, which likely overestimate the association as risk factors and suicide outcomes are typically measured at the same time. In addition, evidence on risk factors is mostly based on psychological autopsy studies, relying on family members and friends for background information that might be prone to measurement error and recall bias [8,9]. Prospective research has been very limited and mostly from selected samples based on patient characteristics, specific age groups, and particular regions [10,11]. Studies have also mainly focused on sociodemographic and lifestyle correlates, rather than longitudinal associations between psychiatric factors and suicide outcomes [12]. Despite these limitations in the research to date, sociodemographic factors such as rural residence and poverty and stressful life events have been linked to an increased risk of suicide.

To address these uncertainties, we have examined a broad range of risk factors for suicide and possible suicide from a large prospective study, the China Kadoorie Biobank (CKB), of 0.5 million adults across China.

## Methods

### Study population

We used data from CKB, a nationwide population-based prospective cohort of 512,715 participants. Individuals aged 30 to 79 years were recruited between 2004 and 2008 from 1,175 local communities across 10 geographically defined regions (5 rural and 5 urban) across China. The study sites were selected carefully with the aim of maximizing geographic diversity (including northern and southern regions with very different climates), social diversity (including affluent coastal cities and impoverished inland and rural areas), and prevalence of disease patterns, while taking into account of population stability, geographic area, quality of death and disease registries, and local commitment and capacity [13].

In each area, residents were identified through local residential records and invited to participate in the study by letter and information leaflet of the study. Overall, about 512,715 (33% rural, 27% urban areas) of those invited individuals (1,801,200 registered permanent residents) participated. Nonparticipants were mainly those who were absent from home or reluctant to spend time to visit the local assessment center according to anecdotal reports by field staff. About a third of the nondisabled invitees living in the study areas participated. All participants provided written informed consent. Baseline survey was conducted at local centers set up for the CKB project. Trained interviewers conducted a standardized questionnaire using a laptop-based direct data-entry system. Individuals were followed up after the baseline measurements for a range of outcomes. Ethics approval was obtained from the Oxford Tropical Research Ethics Committee, University of Oxford, United Kingdom, and the Ethics Review Committee of the Chinese Centre for Disease Control and Prevention, Beijing, China. The latter acted as the central ethics review board for the 10 survey sites in this cohort study. More details about the study procedure have been reported elsewhere [14]. A prospectively developed study protocol for this study can be found in the Supporting information file (S1 Protocol).

### Data collection at baseline

As part of a face-to-face interview, we collected information on age, gender, residential location (rural and urban), low income (< = 10,000 Yuan/year), low education (< = 6 years of education), single status (widowed, separated/divorced, never married), and living alone (household size = 1).

In addition, participants were questioned about 3 lifestyle indicators (drinking, smoking, and physical activity). For the assessment of alcohol consumption and smoking, participants were asked whether drinking had led to problems at work, depression or irritation after drinking, or shakes on stopping, and smoked tobacco at the time of the survey, and those who had not smoked weekly were asked if there was a period of at least a year prior to that when they had smoked some tobacco on most days or daily. Participants were categorized as ever-regular smoker or not. Total physical activity was converted into metabolic equivalent hours per day (MET-hours/day) spent on work, transportation, housework, and nonsedentary recreation. Low physical activity was defined as MET less than 10 hours per day. Body mass index (BMI) was calculated based on the measures of height and weight of participants. Three categories were made: BMI < 18.5, BMI between 18.5 and 25, and BMI larger than 25 according to WHO cut-off scores [15].

In relation to stressful life events, participants were asked: “Over the past two years have you had any of the following major events in your life?” followed by a range of life events, such as marital separation/divorce, major conflict with family, bankruptcy, loss of job, and illness of relative. These were divided into 3 categories including family-related events, finance-related events, and family mental disorder diagnoses, as previous studies have suggested differential associations among these categories with suicide risk [16].

For major physical illnesses, respondents were asked: “Has a doctor ever told you that you had the following disease?” followed by a list of diseases including diabetes, coronary heart disease (CHD), stroke or transient ischemic attack (TIA), hypertension, rheumatic heart disease, tuberculosis, emphysema/bronchitis, asthma, cirrhosis/chronic hepatitis, peptic ulcer, gallstone/gallbladder disease, kidney disease, fracture, rheumatoid arthritis, neurasthenia, head injury, and cancer. Participants reported having one or more of these illnesses were classified as having major physical illness. Under the same question, psychiatric disorders were included. If the answer is yes, participants were asked to indicate whether they are still on treatment. Individuals who were still in treatment were classified as having a current psychiatric disorder.

Depressive disorder and generalized anxiety disorder were assessed using the Chinese version of the computerized Composite International Diagnostic Inventory–Short Form (CIDI-SF) using face-to-face interviews by trained health workers at the study clinic. The CIDI is a fully structured diagnostic instrument based on criteria from the Diagnostic and Statistical Manual of Mental Disorders-IV (DSM-IV) and has moderate concordance with clinical psychiatric interviews [17,18]. The Chinese version of the CIDI produces similar population estimates of depressive disorder to the Structured Clinical Interview for DSM (SCID) [19,20]. Depression was defined by the presence of dysphoria and/or anhedonia for more than 2 weeks during the past 12 months and accompanied by at least 3 of 7 following symptoms, including weight or appetite change, sleeping problems, psychomotor changes, fatigue, concentration problems, feelings of guilt or worthlessness, and thoughts of suicide [21]. Generalized anxiety disorder was indicated by the presence of excessive anxiety or worry for at least 6 months accompanied by the presence of irritability, muscle tension, sleep disturbances, difficulty concentrating, tiring easily, and feelings of restlessness. Data on schizophrenia-spectrum disorders were obtained by linkage to national health insurance hospital admission records. These disorders were diagnosed in hospital and coded in International Classification of Diseases, 10th version (ICD-10, F20-F29) [22].

For the measurement of sleep disorders, participants were requested to answer “yes” or “no” to whether they had experienced 3 forms of sleep disturbance in the past month. If they reported “having trouble falling asleep (sleep onset latency ≥ 30 min) after going to bed or waking up in the middle of the night at least 3 days a week,” they were classified as having disorders of initiating and maintaining sleep (DIMS); those reporting “waking up too early and not be able to get back to sleep at least 3 days a week” were classified as having disorders of early morning awakening (EMA); those who reported “having trouble keeping sober-minded during day time because of bad sleep at least 3 days a week” were classified as having daytime dysfunction (DDF); and those reporting one or more of the 3 aforementioned disorders were classified as any sleep disorders.

We also included a subjective rating of physical health and satisfaction in life. Subjective feelings of physical health was measured with question “How is your current general health status?” and indicated with 4 options (excellent, good, fair, and poor). Participants were divided into poor health and the rest. For satisfaction in life, participants were asked “In general, how satisfied are you with your life?” and to indicated one of the answers: very satisfied, satisfied, neither satisfied nor dissatisfied, unsatisfied, and very unsatisfied. Participants were divided into 2 groups (unsatisfied, including those answered unsatisfied and very unsatisfied, and the rest).

### Measurement of outcomes

Participants were followed up from the baseline measurement (2004 to 2008) until 31 December 2016 through the linkage to China’s Disease Surveillance Points system [23], with annual active confirmation of survival through local residential and administrative records. The Disease Surveillance Points system provides reasonably complete and reliable death registration. Almost all adult deaths were medically certified. For the few (<5%) without medical attention prior to death, standardized procedures were used to determine probable causes of death from symptoms or signs described by relevant informants, usually family members [24]. The trained Disease Surveillance Points system staff coded all diseases on the death certificate and assigned underlying causes using the ICD-10. Suicide deaths were identified using ICD-10 codes X60-84 and Y10-34 [25].

As suicides tend to be undercounted [26], in addition to commonly used ICD-10 codes for suicide, we also used a range of ICD codes to identify possible suicide in the Chinese context, including poisoning (T39.0, T39.1, T39.3, T40, T42, T43), toxic effects of substances such as alcohol, carbon dioxide, and other gas and drugs (T50.9, T51, T52, T58, T59.7, T59.8, T59.9, T60, W84, X09), sequelae of intentional self-harm (Y87.0), personal history of self-harm (Z91.5), falling from high places such as cliff and buildings (W13, W15, W16, W17, W19), and drowning (W65-W74). All accidental events were excluded as possible suicide.

### Statistical analyses

We conducted Cox regression model to estimate the associations between risk factors and suicide outcomes. We adopted Cox regression to control for time-to-event and account for potential impact of other causes of death as a competing event of death by suicide. That is, instead of omitting people who died during follow-up from the survival analyses, we treated “failure” from other causes of death as a censored observation, while “failure” from the outcome of interest (e.g., death by suicide) as an event. We did not use weighting in our analyses, instead, we adjusted for a range of sociodemographic factors, including gender, age, residence, income, education, and single status, except for living alone which was strongly linked to single status [φ = .52, *p* < 0.001]). They were included in the regression models as binary or categorical variables.

We did a series of additional analyses. First, we controlled for risk factors in other domains in addition to sociodemographic factors. When investigating effects of lifestyle factors, we controlled for sociodemographic factors; when testing effects of physical health status, we additionally adjusted for effects of lifestyle factors; when testing effects of mental health status, we controlled for sociodemographic factors, lifestyle factors, as well as major physical illnesses. As sleep disturbance is a common symptom of psychiatric illness [27], we also adjusted for current psychiatric disorders when assessing the association between sleep disorders and suicide risk. Second, we compared whether effects of risk factors in all domains differed by gender (women versus men) and residence status (rural versus urban). We made changes to the original analytical plan by conducting additional analyses using age and BMI as continuous variables to provide more information on their association with suicide death. Third, to test the robustness of the findings, we ran sensitively analyses by examining the association between all examined risk factors and possible suicide. All Cox regression models were conducted within STATA version 13. This study is reported as per the Strengthening the Reporting of Observational Studies in Epidemiology (STROBE) and The REporting of studies Conducted using Observational Routinely-collected health Data (RECORD) guidelines (S1 The RECORD Checklist).

## Results

Among the 512,715 participants at baseline, the mean age at baseline was 52 (SD = 10.7), 59% were female, 55.9% lived in rural areas, and 9.4% were single. Around 15% reported having physical illnesses and prevalence of mental disorders ranged from 0.2% to 16.8% (Tables 1 and 2).

**Table 1:** Demographic and lifestyle characteristics and stressful life events of participants by suicide status during follow-up.

	Suicide (*n =* 520)	Possible suicide (*n* = 554)	No suicide (*n* = 511,641)	All (*n* = 512,715)
**Sociodemographic factors**				
Age group (median age) *N* (%)				
30–39	(36.8^a^) 40 (7.7%)	(37.7) 42 (7.6%)	(37.4) 15.2%	(37.4) 15.1%
40–49	(44.4) 106 (20.4%)	(43.4) 116 (20.9%)	(44.5) 29.8%	(44.5) 29.8%
50–59	(55.1) 165 (31.7%)	(55.3) 161 (29.1%)	(54.5) 30.7%	(54.5) 30.7%
60–69	(65.3) 144 (27.7%)	(65.4) 145 (26.2%)	(64.6) 17.9%	(64.6) 17.9%
70–79	(72.9) 65 (12.5%)	(72.5) 90 (16.2%)	(72.3) 6.4%	(72.3) 21.6%
Female (%)	253 (48.7%)	212 (38.3%)	302,045 (59.0%)	302,510 (59.0%)
Rural residence (%)	425 (79.8%)	424 (76.5%)	285,694 (55.8%)	286,533 (55.9%)
Low education (<6 years)	381 (73.3%)	379 (68.4%)	259,596 (50.7%)	260,356 (50.8%)
Income (<¥10,000 [€1,300])	230 (44.2%)	232 (41.9%)	144,272 (28.2%)	144,734 (28.2%)
Single	96 (18.5%)	108 (19.5%)	48,047 (9.4%)	48,251 (9.4%)
Living alone	39 (7.5%)	34 (6.1%)	14,476 (2.8%)	14,549 (2.8%)
**Lifestyle factors**				
Problem drinking				
Male	19 (7.1%)	31 (9.1%)	12,807 (6.1%)	12,857 (6.1%)
Female	0 (0%)	1 (0.5%)	466 (0.2%)	467 (0.2%)
Ever-regular smoker				
Male	195 (73.0%)	276 (80.7%)	155,811 (74.3%)	12,857 (74.3%)
Female	10 (4.0%)	12 (5.7%)	9,776 (3.2%)	9,798 (3.2%)
Low physical activity (MET <10)	171 (32.9%)	175 (31.6%)	121,218 (23.7%)	121,564 (23.7%)
**Stressful life events**				
Family-related events	25 (4.8%)	20 (3.6%)	10,246 (2.0%)	10,291 (2.0%)
Finance-related events	7 (1.3%)	2 (0.4%)	5,790 (1.1%)	5,961 (1.2%)
Family member with mental disorder diagnosis	10 (1.9%)	12 (2.2%)	9,442 (1.8%)	9,464 (1.8%)

^a^Median years of age.

MET, Metabolic Equivalent Task.

**Table 2:** Physical and mental health status of participants by suicide status during follow-up.

	Suicide (*n* = 520)	Possible suicide (*n* = 554)	No suicide (*n* = 511,641)	All (*n* = 512,715)
**Physical health factors**				
BMI (<18.5)	59 (11.3%)	138 (24.9%)	22,248 (4.3%)	22,361 (4.4%)
18.5≤BMI≤25	349 (67.1%)	362 (65.3%)	320,728 (62.7%)	321,439 (62.7%)
BMI (>25)	112 (21.5%)	54 (9.7%)	168,663 (33.0%)	168,913 (32.9%)
Current physical illnesses*	121 (23.3%)	102 (18.4%)	79,922 (15.6%)	80,145 (15.6%)
Poor physical health	110 (21.2%)	79 (14.3%)	52,900 (10.3%)	53,089 (10.4%)
**Mental health factors**				
Depression disorders	10 (1.9%)	2 (0.4%)	3,342 (0.7%)	3,354 (0.7%)
Anxiety disorders	4 (0.8%)	1 (0.2%)	1,273 (0.2%)	1,278 (0.2%)
Schizophrenia-spectrum disorders	21 (4.0%)	12 (2.2%)	1,295 (0.3%)	1,328 (0.3%)
Sleep disorder	132 (25.4%)	110 (9.9%)	85,858 (16.8%)	86,100 (16.8%)
Psychiatric disorders (lifetime)	17 (3.3%)	11 (2.0%)	1,878 (0.4%)	1,906 (0.4%)
Psychiatric disorders (current)	13 (2.5%)	7 (1.3%)	860 (0.2%)	860 (0.2%)
Unsatisfied with life	29 (5.6%)	20 (3.6%)	21,283 (4.2%)	21,332 (4.2%)

BMI, Body Mass Index; the two categories ‘Psychiatric disorders’ are self-reported.

**Fig 1:**
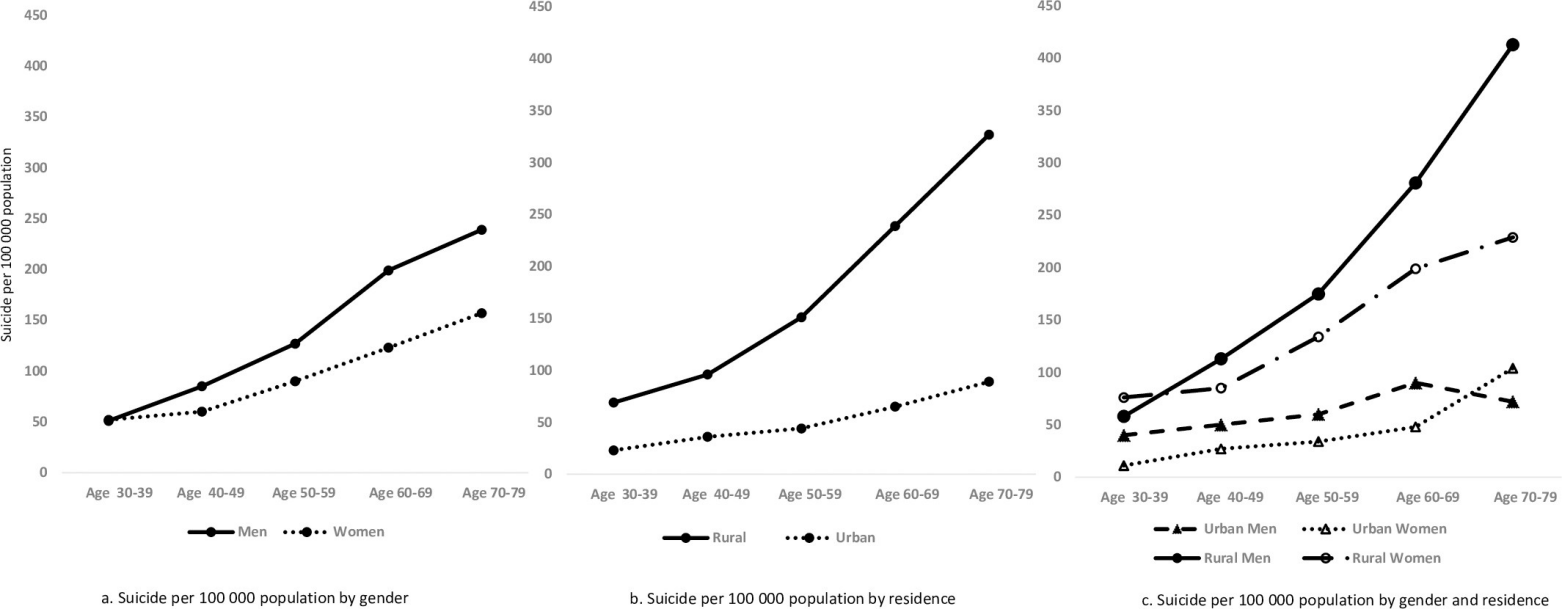
Mortality rates of suicide by gender, residence, and age group. Fig 1a. Suicide per 100,000 population by gender; Fig 1b. Suicide per 100,000 population by residence; Fig 1c. Suicide per 100,000 population by gender and residence.

During the average follow-up of 9.9 years, there were 520 deaths by suicide (at a rate of 102 suicides per 100,000 persons or 10.2 per 100,000 annually), and 51.3% were in men. Around 70% of suicide cases in individuals with lower education and 78% from rural residential areas. The suicide mortality rates increased with age and were higher in rural than urban areas (145 versus 46 per 100,000) and in men than women (127 versus 84 per 100,000) (Fig 1). The rate of possible suicide was 108 per 100,000 (*n =* 554). Similar demographic patterns were found for possible suicide by areas of residence (148 versus 58 per 100,000, in rural and urban, respectively) and between males (163 per 100,000) and females (70 per 100,000) (S1 Fig).

**Fig 2:**
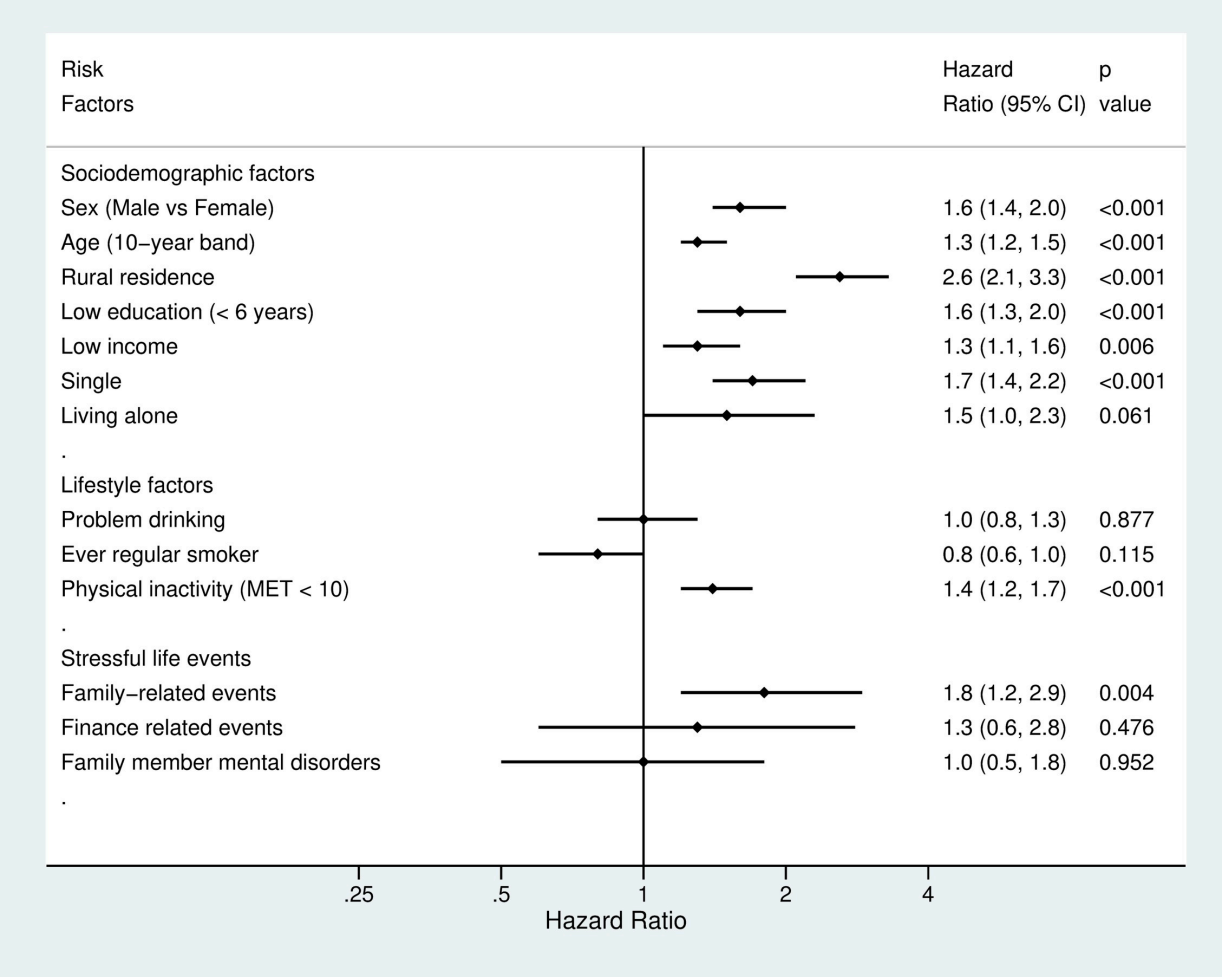
Hazard ratios for suicide by sociodemographic and lifestyle factors and stressful life events. HRs adjusted for sex, age, rural residence, education, income, and single status. HR, hazard ratio; MET, Metabolic Equivalent Task.

**Fig 3:**
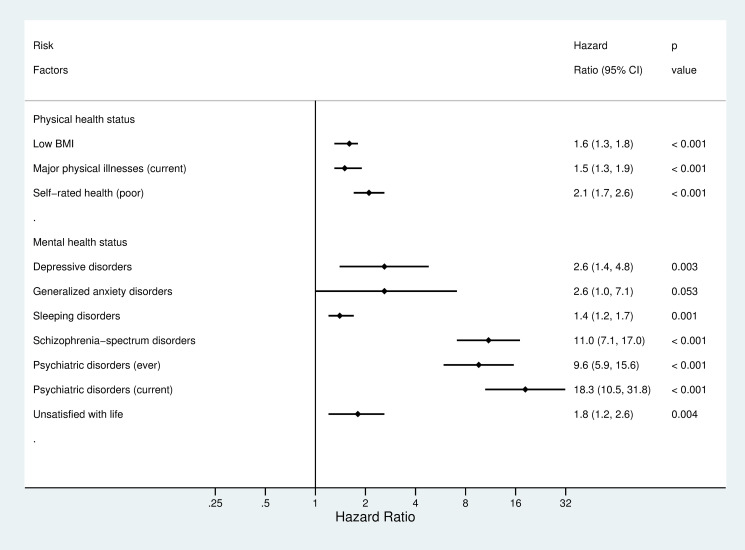
Hazard ratios for suicide by physical and mental health status. HRs adjusted for sex, age, rural residence, education, income, and single status. HR, hazard ratio; BMI, body mass index.

All risk factors in the domain of physical health status, all but 1 risk factor in each of 2 domains of sociodemographic factors and mental health status, and 1 risk factor in the other 2 domains, lifestyle factors and stressful life events, were associated with an increased risk of suicide (Figs 2 and 3). After adjustment for sociodemographic factors, HRs for suicide ranged from 1.3 (95% CI: 1.1 to 1.6, *p* = 0.004) for low-income status to 2.6 (2.1 to 3.3, *p* < 0.001) for rural residence. In addition, subgroup analyses indicated that there were no clear differences in the link between income and suicide risk by residential areas, which was 1.5 (0.9 to 2.5, *p* = 0.091) in urban areas versus 1.3 (1.0 to 1.5, *p* = 0.016) in rural areas. Higher age band was associated with a higher risk of suicide (1.3 [1.2 to 1.5], *p* < 0.001). Age was associated with suicide risk when it was entered to the regression model as a continuous variable (B = 0.03, SE = 0.01, *p* < 0.001, HR = 1.03 [1.02, 1.04]), indicating that 1 year increase in age was associated with a 3% higher risk of suicide. Sensitivity test of nonlinearity of the age–suicide link did not indicate a better model than the linear model (df = 1, χ^2^ = 0.18, *p* = 0.67). Adjusted hazard ratios (aHR) for suicide ranged from 0.8 (0.6 to 1.0, *p* = 0.115) for ever having been regular smoker to 1.4 (1.2 to 1.7, *p* < 0.001) for physical inactivity among lifestyle factors. In addition, when BMI was entered into the model as a continuous variable, 1 unit of increase in BMI score was associated with a 7% lower risk of suicide (B = −0.07, SE = 0.01, *p* < 0.001, HR = 0.93 [0.91, 0.96]). A lower BMI was associated with a higher risk of suicide (1.6 [1.3 to 1.8, *p* < 0.001]) when BMI was categorized according to WHO cut-off scores. Hazards were increased for family-related stressful life events (aHR = 1.8 [1.2 to 2.9, *p* = 0.004]). However, there were no clear links between suicide and other type of stressful life events. In relation to physical health, aHR varied from 1.5 (1.3 to 1.9, *p* < 0.001) for current major illnesses to 2.1 (1.7 to 2.6, *p* < 0.001) for self-reported poor physical health. For individual mental disorders, aHR ranged from 1.4 (1.2 to 1.7, *p* = 0.001) for sleep disorders to 11.0 (7.1 to 17.0, *p* < 0.001) for schizophrenia-spectrum disorders.

We additionally controlled for risk factors in other domains in addition to sociodemographic factors. After additionally adjusting for lifestyle factors including weekly drinking, regular smoking, and physical inactivity, aHR was 1.5 (1.2 to 1.9, *p* < 0.001) for major physical illnesses and 2.0 (1.6 to 2.5, *p* < 0.001) for poor self-reported physical health. aHR varied from 1.2 (1.0 to 1.4, *p* = 0.020) for sleep disorders to 10.6 (6.8 to 16.5, *p* < 0.001) for schizophrenia-spectrum disorders. There were no material changes between HRs with and without additional adjustments. In addition, after adjusting for current psychiatric disorders, the association between sleep disorders and increased suicide risk remained (aHR = 1.2 [1.0 to 1.4, *p* = 0.021]). Finally, we compared the associations between risk factors and suicide by gender and residence. There were no differences by gender or residence in the magnitude of the link between risk factors and later suicides (S1 Table).

In a further sensitivity analysis, we investigated risk factors for possible suicide (*n =* 554). Overall, the effect was similar for sociodemographic, physical health, and lifestyle domains but not for all mental health variables (S2 and S3 Figs). Several specific psychiatric disorders were not associated with possible suicide, including depression (aHR = 0.5 [0.1, 2.0], *p* = 0.324), generalized anxiety disorders (aHR = 0.7 [0.1, 4.7, *p* = 0.681]), and sleep disorders (aHR = 1.1 [0.9, 1.3, *p* = 0.547]) (S3 Fig).

## Discussion

In this prospective study of 512,715 individuals from 10 geographical regions in China, we examined suicide rates and risk factors. In total, 520 individuals died by suicide during an average follow-up period of 9.9 years (10.1 per 100,000 persons annually), comparable to the global average rate (10.5 per 100,000 persons annually) [28]. We found that the risk of suicide was higher in men than women, and those from rural areas had a 3 times higher suicide risk than urban dwellers. Among examined risk factors, mental health factors were associated with the highest suicide risks.

The new work adds to the evidence base in the following ways. First, we have shown that diagnosed mental disorders were associated with a higher risk of suicide. Overall, though, the relative risks of mental disorders were lower than that reported in high-income countries, consistent with the finding of lower absolute prevalence of mental disorders in suicide [29]. For example, we estimated an 11-fold increased risk of suicide in those with a schizophrenia-spectrum disorder when compared to those without this disorder, while in a population-based cohort study in Sweden, the reported risk of suicide in the same diagnostic group was 20 times higher [30], similar to a meta-review of risk factors for suicide [31]. Previous work has reported a HR of 9 for individuals with depressive disorders in a meta-analysis of 35 studies [32], higher than the increased hazard of 2.6 in the current study.

The underdiagnosis of mental disorders due to stigma in China might explain this discrepancy [33,34]. For instance, the rate of depression was 0.7% in this cohort, lower than a rate of 1.7% in a WHO survey using CIDI in Beijing and Shanghai [35]. In this cohort study, individuals with and without mental disorders were followed up for 10 years for their suicide outcomes. If mental disorders were underdiagnosed, some of the individuals who died by suicide would be categorized as without mental disorders when they did have mental disorders. In this scenario, hazards of suicide in individuals without mental disorders would be overestimated, and the ratio of the hazard risk of suicide among individuals with a mental disorder to the risk of suicide among individuals without a mental disorder would be underestimated. This suggests that our risk estimates are likely conservative, and future work is required to replicate these findings. Another explanation for this discrepancy could lie in the study design. Most studies examining the link between mental disorders and suicide risk have been case-control psychological autopsy studies in which typically a psychiatric diagnosis directly prior to suicide death was examined [32]. Longer follow-ups have been associated with lower hazards for mental disorders. Our findings are more consistent with a cohort study which had a 10-year follow-up and used DSM-criteria for depressive disorders (HR = 1.8 [1.7 to 2.0]) [36]. Nevertheless, the lower relative risk for mental disorders in China compared to other countries might be a real finding. At the same time, mental disorders still had the highest risk estimates among the examined risk factors. Thus, improving access to mental health services and providing evidence-based assessment and treatment is one clear implication of these findings.

A second novel finding is the contribution of sleep disorder. We found that sleep disorders were modestly associated with an elevated risk of suicide. This is consistent with the findings of prospective investigations in other Asian countries [37,38], and one Norwegian study [39]. Sleep problems are highly prevalent, affecting 10% to 40% of the general population [40,41] and consistent with this the current study reported a rate of 26%. The increased risk of suicide in this group suggests that clinical assessment of suicide risk among individuals with persistent sleep problems might be beneficial for suicide prevention in China.

Third, we found that suicide rates were higher in rural areas, which is consistent with previous research in China [42,43]. The higher rate might be due to lower availability of effective interventions after a suicide attempt and more ready access to lethal means of suicide such as hazardous pesticides [44]. Thus, even if one assumes the same rate of suicide attempts, suicide death might be more likely to occur in the rural areas as medical assistance is less accessible in emergencies, and the overall quality of medical services is poorer [45]. In support, a psychological autopsy study of 895 suicide victims in China reported that 61% of suicide deaths were associated with unsuccessful medical resuscitation [46]. Other explanations for the higher suicide rate in the rural areas could be selection effects as individuals with a higher education, less underlying psychiatric morbidity, and more economic resources move to cities. The higher risk reported in rural areas is also reported in some high-income countries [47–49]. These findings suggest targeted approaches in rural populations could be considered in national suicide prevention efforts. Further research into risk factors of rural suicide is required [50].

Although suicide risk was higher in rural than urban areas, the groups contributing to these differences have changed over the last 20 to 30 years. Previously, young women in rural areas were at high risk [6]. However, economic and social changes have provided women with increased opportunities in education and work and provided more independence including financially [51]. The change to higher risk in men has been shown in previous studies [42,43], and the earlier excess in women in the 1990s [12] has not been found since. In the current study, rural men between 70 and 79 years old had the highest suicide risks, with a rate of 413 per 100,000 population during the follow-up of 9.9 years. Suicide risk in elderly might be linked to loneliness and lack of social support network [52,53], and we found that 20% of suicide decedents were living alone and that 46% were single in those aged 70 to 79 years, compared to less than 5% living alone and 15% single in those aged 30 to 59 years old. Limited data has suggested some differences in suicide method by residence. For instance, in this study, pesticide was used more often in rural areas as a suicide method (9/415, 2%) than in urban areas (1/145, 1%). Future research is needed to examine this.

A final finding of the current study is that individuals with a major physical illness had a 50% increased risk of suicide. This estimate is lower than case-control studies in China which reported a 3-fold higher risk among individuals with physical illness than matched controls [54]. Chronic physical illnesses were more prevalent in the older people. In this study, prevalence was 3.9% in those aged 30 to 39 years but 32.0% in the 70 to 79 age group. However, the effects of physical illness on suicide risk were similar between age groups and residential areas. The effects of specific physical illnesses needs further research [55], a finding consistent with research in high-income settings [56].

Family-related events, such as separation or divorce, family conflict, and death of a spouse or illness were linked to a higher risk of suicide. However, financial stressors, including loss of income, bankruptcy, and loss of job or retirement, were not clearly associated. This suggests that family-related events had stronger associations with suicide than financial concerns. This finding is similar to research on suicidal behaviors in Chinese adolescents, which suggests that problems in family relationships are important to consider in risk assessment and management [16]. As a consequence, the role of family-based interventions on reducing suicide thoughts and behaviors [57] needs examination in China. Furthermore, data from the same cohort also reported that, compared to finance-related stressful life events, family-related stressful life events had a higher correlation with other negative outcomes such as depression [58]. Nevertheless, in this study, financial stress was defined as experiencing loss of job or income or bankruptcy over the past 2 years. Longer-term erosion of economic opportunity, including the perceived prospects of poor occupation status [59], unemployment [60], and low salary [61], have been linked to increased suicide risk. These findings are consistent with the elevated risks observed for rural residents and men who were unsatisfied with life.

Many risk factors associated with suicide were also linked to possible suicide, although in general with lower effect sizes. However, there were large differences in mental health factors, where common disorders including depression and generalized anxiety disorders were not associated with possible suicide. The effects of any psychiatric disorder and schizophrenia-spectrum disorders on possible suicide were also considerably smaller. These differences in the effect of psychiatric risk factors suggests that possible suicide and suicide (that includes undetermined deaths) as defined in this study might belong to different phenotypes. This underscores that our decision to classify possible suicide separately was justified, and further work will need to clarify whether these deaths are more accurately classified as accidents. It is also possible that evidence of mental health is one of the factors which contribute to a death being determined as suicide, especially as there is limited information from coroners in China in determining suicide death [62]. If there is a background of mental illness, then it is possible that in uncertain cases, these are more likely to be classified as suicide rather than possible suicide in China. This may explain the lack of an association between some mental disorders and possible suicide, and the overall weaker associations between them than the link between mental health and suicide death.

Our study has several strengths. First, we conducted the largest cohort study to date of suicide using data from more than half a million individuals in China with a broad geographic and social diversity. Therefore, the findings are generalizable to the general Chinese population. Second, by using a prospective design, we compared individuals with and without the examined risk factors on later suicide outcomes during follow up. This helps to mitigate recall bias which is often present in retrospective studies [9] and provide a more precise estimation of the associations between risk factors and suicide than cross-sectional and psychological autopsy studies. Third, we used structured clinical assessments and self-report data in the baseline measurement, unlike many earlier psychological autopsy studies in which information on risk factors were from other informants who might not have reliable or sufficiently detailed knowledge of the individuals who died by suicide. In addition, the proxy informants and the interviewers in previous autopsy studies were also aware of the reported cause of death being suicide, which could introduce report bias. Thus, our study design allowed for a more reliable measurement of risk factors.

Some limitations have to be noted. First, we cannot infer a causal relationship between identified risk factors and suicide. However, the studied variables were independently linked to the suicide risk, as we adjusted for a wide range of potential confounders. Second, we only examined the effects of a limited number of mental disorders. Other conditions, such as bipolar disorder, personality disorders, eating disorders, and substance use disorders, have been associated with increased suicide risk [31,63]. Future research on the link between these mental disorders and suicide is needed. Third, due to lack of data, we were not able to examine attempted suicide, as it appeared to be a strong risk factor for suicide death [64]; future study as a risk factor as well as an outcome is recommended. Fourth, the studied cohort were aged from 30 to 79 years at baseline measurement. Thus, findings may not be applicable to younger populations, such as adolescents, where suicide rates are relatively low. In addition, 33% of invited individuals visited the local assessment center to participate in this study. As sociodemographic data of individuals who did not participate in the study were not collected, it is not possible for us to know how well the cohort could be generalized to the general population. However, it is possible to compare the included sample with general population based on information from other official sources. This suggests differences in sex distribution but similar in terms of urban versus rural residence. Compared to the general population during the baseline measurement years (2004 to 2008) in China [65,66], the Kadoorie cohort had a slightly higher percentage of female (59.0% versus 48.6%) but a similar residence distribution (55.9% versus 54.0% rural). Furthermore, income at separate time periods might play a different role on suicide risk, and future studies could examine this. Finally, although CIDI is a validated diagnostic instrument, the face-to-face interview format and stigma toward mental illnesses in China might be associated with underreporting of mental disorders in this population. If this is the case, then our reported links with mental disorders are conservative estimates.

In summary, we followed up over half a million individuals for suicide for an average of 10 years. We prospectively examined the relationships between a range of risk factors, including sociodemographic, lifestyle, physical and mental health, and stressful life events, and suicide outcomes. Almost all of the studied risk factors were associated with increased hazards of suicide death. Men and individuals from rural areas had higher suicide risks, and we identified rural elderly men as a high-risk group. Mental disorders as a category were associated with the highest suicide risk. This pattern and magnitude of risk factors should be considered as part of suicide prevention strategies in China.

## Supporting information

S1 The RECORD Checklist Checklist of items, extended from the STROBE statement, that should be reported in observational studies using routinely collected health data. (DOCX)

S1 Protocol Risk factors for suicide among 0.5 million Chinese adults. (DOCX)

S1 Table Hazard ratios for suicide by sociodemographic factors, lifestyle factors, stressful life events, physical and mental health status for male and female separately. (DOCX)

S2 Table Hazard ratios for suicide by sociodemographic factors, lifestyle factors, stressful life events, physical and mental health status for rural and urban residence separately. (DOCX)

S3 Table Crude hazard ratios for suicide and possible suicide by sociodemographic factors, lifestyle factors, stressful life events, physical and mental health status. (DOCX)

S1 Fig Mortality rates of possible suicide by gender, residence, and age group. (TIF)

S2 Fig HRs for possible suicide by sociodemographic factors, lifestyle factors, and stressful life events. HRs adjusted for sociodemographic factors including sex, age, rural residence, education, income, and single status. HR, hazard ratio; MET, Metabolic Equivalent Task.
(TIF)

S3 Fig HRs for possible suicide by physical and mental health status. HRs adjusted for sociodemographic factors including sex, age, rural residence, education, income, and single status. HR, hazard ratio; BMI, body mass index.
(TIF)

## Abbreviations

aHR: adjusted hazard ratio
BMI: body mass index
CHD: coronary heart disease
CIDI-SF: Composite International Diagnostic Inventory–Short Form
CKB: China Kadoorie Biobank
DDF: daytime dysfunction
DIMS: disorders of initiating and maintaining sleep
DSM-IV: Diagnostic and Statistical Manual of Mental Disorders-IV
EMA: early morning awakening
HR: hazard ratio
ICD-10: International Classification of Diseases, the 10th version
MET-hours/day: metabolic equivalent hours per day
RECORD: REporting of studies Conducted using Observational Routinely-collected health Data
SCID: Structured Clinical Interview for DSM
STROBE: Strengthening the Reporting of Observational Studies in Epidemiology
TIA: transient ischemic attack
