# Supplementary material for: Factors associated with suicide risk among Chinese adults: A prospective cohort study of 0.5 million individuals
Source: PLoS Med. 2021 Mar 11;18(3):e1003545. doi: 10.1371/journal.pmed.1003545 (PMC7951865; doi:10.1371/journal.pmed.1003545)
Supplement: S1 Protocol — (DOCX) [file pmed.1003545.s002.docx]

**Risk factors for suicide among 0.5 million Chinese adults**

Dr. Rongqin Yu, Dr. Yiping Chen, Prof. Dr. Zhengming Chen and Prof. Dr. Seena Fazel, University of Oxford

**Background and Objective:** Suicide in China accounts for about a quarter of all suicides worldwide. It is ranked as the fifth leading cause of death for the general population in China. However, the pattern of suicide (including completed suicide and suicide attempts) and their risk factors remain unknown. The aim of the current study is to examine epidemiological patterns and risk factors for suicide and suicide attempts among a representative sample of 0.5 million Chinese adults. **Design and Method:** We proposal a study using data from the Kadoorie cohort (1). The exposure variables include depressive symptoms, stressful life events (including family-related events, finance-related events, or violence victimization), and living situation (living alone or cohabit), religion, health issues (chronic illness and general poor health), and drug and alcohol use. Depression symptoms are measured by the Chinese version of the WHO 12-month Composite International Diagnostic Interview short-form for depression, which has shown good validity (2). Data on major stressful life events experienced over the past 2 years, drug/alcohol use, chronic illness, and other factors are collected by trained health workers in a structured interview. As for outcomes, information on suicide death will be retrieved from official death registries. Information on possible suicide will be gathered from health insurance records. Logistic regression will be performed to assess the association between a range of risk factors and suicide and possible suicide. We will take into account potential effects of confounders such as sex, education, occupation, rural/urban residence, socio-economic status, and prior history of psychiatric disorders. This study will provide important new information into risk factors of suicide and possible suicide.

1. Chen Z, Chen J, Collins R, Guo Y, Peto R, Wu F, et al. China Kadoorie Biobank of 0.5 million people: survey methods, baseline characteristics and long-term follow-up. Int J Epidemiol. 2011;40(6):1652-66.

2. Chen Y, Bennett D, Clarke R, Guo Y, Yu C, Bian Z, et al. Patterns and correlates of major depression in Chinese adults: a cross-sectional study of 0.5 million men and women. Psychol Med. 2017;47(5):958-70.
