# Supplementary material for: Factors associated with suicide risk among Chinese adults: A prospective cohort study of 0.5 million individuals
Source: PLoS Med. 2021 Mar 11;18(3):e1003545. doi: 10.1371/journal.pmed.1003545 (PMC7951865; doi:10.1371/journal.pmed.1003545)
Supplement: S1 Table — (DOCX) [file pmed.1003545.s003.docx]

Supplementary Table 1. Hazard ratios for suicide by sociodemographic factors, lifestyle factors, stressful life events, physical and mental health status for male and female separately

|  | Suicide | | | | | | | | |
| --- | --- | --- | --- | --- | --- | --- | --- | --- | --- |
|  | Male | | | |  | Female | | | |
|  | cHR (95% CI) | *p* | aHR (95% CI) | *p* |  | cHR (95% CI) | *p* | aHR (95% CI) | *p* |
| **Sociodemographic factors** |  |  |  |  |  |  |  |  |  |
| Age (10-year band) * | 1.6 (1.4-1.7) | <0.001 | 1.4 (1.2-1.5) | <0.001 |  | 1.4 (1.3-1.6) | <0.001 | 1.4 (1.2-1.5) | <0.001 |
| Rural residence | 2.9 (2.1-3.9) | <0.001 | 2.1 (1.5-2.9) | <0.001 |  | 3.3 (2.4-4.5) | <0.001 | 3.1 (2.2-4.4) | <0.001 |
| Low education (< 6years) | 3.2 (2.5-4.2) | <0.001 | 1.7 (1.3-2.3) | <0.001 |  | 2.6 (2.0-3.6) | <0.001 | 1.4 (1.0-1.9) | 0.051 |
| Low income | 2.4 (1.9-3.1) | <0.001 | 1.4 (1.1-1.9) | 0.005 |  | 1.7 (1.4-2.2) | <0.001 | 1.2 (0.9-1.5) | 0.241 |
| Single | 3.6 (2.7-4.8) | <0.001 | 2.3 (1.7-3.2) | <0.001 |  | 1.7 (1.2-2.3) | 0.001 | 1.3 (0.9-1.8) | 0.163 |
| Living alone | 4.4 (2.9-6.7) | <0.001 | 1.5 (0.9-2.6) | 0.149 |  | 2.0 (1.1-3.4) | 0.014 | 1.3 (0.7-2.6) | 0.393 |
| **Lifestyle factors** |  |  |  |  |  |  |  |  |  |
| Problem drinking | 1.2 (0.7-1.8) | 0.534 | 1.1 (0.7-1.7) | 0.707 |  | nc |  | nc |  |
| Ever regular smoker | 0.9 (0.7-1.2) | 0.644 | 0.8 (0.6-1.1) | 0.211 |  | 1.3 (0.7-2.4) | 0.428 | 0.8 (0.4-1.6) | 0.543 |
| Physical inactivity (MET < 10) | 1.7 (1.4-2.2) | <0.001 | 1.5 (1.1-1.9) | 0.006 |  | 1.5 (1.2-2.0) | 0.002 | 1.4 (1.1-1.9) | 0.016 |
| **Stressful life events** |  |  |  |  |  |  |  |  |  |
| Family-related events | 2.5 (1.4-4.8) | 0.004 | 1.5 (0.8-2.8) | 0.258 |  | 2.6 (1.6-4.4) | <0.001 | 2.4 (1.4-4.2) | 0.002 |
| Finance related events | 0.3 (0.4-2.1) | 0.214 | 0.3 (0.0, 2.3) | 0.261 |  | 2.1 (0.9-4.7) | 0.075 | 2.6 (1.2-5.9) | 0.020 |
| Family member mental disorders | 1.0 (0.4-2.3) | 0.936 | 0.9 (0.4-2.2) | 0.816 |  | 1.1 (0.5-2.7) | 0.826 | 1.1 (0.4-2.6) | 0.873 |
| **Physical health status** |  |  |  |  |  |  |  |  |  |
| Low BMI | 2.4 (1.9-3.1) | <0.001 | 1.7 (1.4-2.2) | <0.001 |  | 1.6 (1.2-2.0) | <0.001 | 1.4 (1.1-1.7) | 0.005 |
| Major physical illnesses (current) | 1.8 (1.4-2.4) | <0.001 | 1.6 (1.2-2.2) | 0.001 |  | 1.7 (1.3-2.2) | <0.001 | 1.5 (1.1-2.0 | 0.007 |
| Self-rated poor health | 2.5 (1.9-3.5) | <0.001 | 2.0 (1.5-2.8) | <0.001 |  | 2.5 (1.7-3.3) | <0.001 | 2.1 (1.6-2.8) | <0.001 |
| **Mental health status** |  |  |  |  |  |  |  |  |  |
| Depressive disorders | 2.5 (0.8-7.8) | 0.116 | 1.9 (0.6-6.0) | 0.261 |  | 3.5 (1.7-7.5) | 0.001 | 3.1 (1.4-6.5) | 0.004 |
| Anxiety disorders | 4.5 (1.1-18.0) | 0.035 | 3.5 (0.9-13.9) | 0.082 |  | 2.4 (0.6-9.8) | 0.207 | 2.2 (0.5-8.7) | 0.278 |
| Sleep disorders | 2.0 (1.5-2.7) | <0.001 | 1.6 (1.2-2.2) | 0.001 |  | 1.5 (1.2-2.0) | 0.002 | 1.2 (0.9-1.6) | 0.136 |
| Schizophrenia-spectrum disorders | 9.9 (5.1-19.2) | <0.001 | 9.2 (4.7-18.0) | <0.001 |  | 12.1 (6.8-21.6) | <0.001 | 12.0 (6.7-21.5) | <0.001 |
| Psychiatric disorders (ever) | 16.8 (9.2-30.7) | <0.001 | 14.9 (8.1-27.5) | <0.001 |  | 5.5 (2.4-12.3) | <0.001 | 5.6 (2.5-12.6) | <0.001 |
| Psychiatric disorders (current) | 20.6 (9.7-43.6) | <0.001 | 19.8 (9.2-42.2) | <0.001 |  | 13.0 (5.8-29.2) | <0.001 | 15.7 (7.0-35.4) | <0.001 |
| Unsatisfied with life | 1.5 (0.9-2.4) | 0.134 | 1.9 (1.1-3.1) | 0.016 |  | 1.2 (0.7-2.1) | 0.550 | 1.6 (0.9-2.9) | 0.119 |

Notes: cHR = crude hazard ratio (not adjusted for any covariates). aHR. aHR = adjusted HR. nc, model did not converge as the number of individuals from the rural with anxiety disorders was 0. MET: Metabolic Equivalent Task. BMI: body mass index. Prior physical illnesses included diabetes, CHD, stroke or TIA, hypertension, rheumatic heart disease, TB, emphysema/bronchitis, asthma, cirrhosis/chronic hepatitis, peptic ulcer, gallstone/gallbladder disease, kidney disease, fracture, rheumatoid arthritis, neurasthenia, head injury, and cancer.
