# Supplementary material for: Factors associated with suicide risk among Chinese adults: A prospective cohort study of 0.5 million individuals
Source: PLoS Med. 2021 Mar 11;18(3):e1003545. doi: 10.1371/journal.pmed.1003545 (PMC7951865; doi:10.1371/journal.pmed.1003545)
Supplement: S2 Table — (DOCX) [file pmed.1003545.s004.docx]

Supplementary Table 2. Hazard ratios for suicide by sociodemographic factors, lifestyle factors, stressful life events, physical and mental health status for rural and urban residence separately

|  | Suicide | | | | | | | | |
| --- | --- | --- | --- | --- | --- | --- | --- | --- | --- |
|  | Rural | | | |  | Urban | | | |
|  | cHR (95% CI) | *p* | aHR (95% CI) | *p* |  | cHR (95% CI) | *p* | aHR (95% CI) | *p* |
| **Sociodemographic factors** |  |  |  |  |  |  |  |  |  |
| Sex (Male vs Female) | 1.5 (1.2-1.8) | <0.001 | 1.6 (1.3-1.9) | <0.001 |  | 1.7 (1.2-2.5) | 0.005 | 2.1 (1.4-3.1) | <0.001 |
| Age (10-year band) * | 1.6 (1.5-1.7) | <0.001 | 1.4 (1.2-1.5) | <0.001 |  | 1.4 (1.2-1.7) | <0.001 | 1.3 (1.1-1.5) | 0.011 |
| Rural residence |  |  |  |  |  |  |  |  |  |
| Low education (< 6years) | 2.3 (1.8-2.9) | <0.001 | 1.7 (1.3-2.2) | <0.001 |  | 1.7 (1.1-2.5) | 0.008 | 1.3 (0.9-2.1) | 0.191 |
| Low income | 1.5 (1.2-1.8) | <0.001 | 1.3 (1.0-1.5) | 0.016 |  | 2.1 (1.3-3.3) | 0.001 | 1.5 (0.9-2.5) | 0.091 |
| Single | 2.5 (1.9-3.2) | <0.001 | 1.6 (1.3-2.1) | <0.001 |  | 2.4 (1.5-3.8) | <0.001 | 2.1 (1.2-3.4) | 0.005 |
| Living alone | 3.4 (2.3-5.1) | <0.001 | 1.3 (0.8-2.2) | 0.262 |  | 4.0 (2.3-7.1) | <0.001 | 2.1 (0.9-4.8) | 0.098 |
| **Lifestyle factors** |  |  |  |  |  |  |  |  |  |
| Problem drinking | 1.2 (0.7-2.0) | 0.442 | 1.0 (0.6-1.7) | 0.973 |  | 1.6 (0.5-5.0) | 0.429 | 1.3 (0.4-4.0) | 0.697 |
| Ever regular smoker | 1.2 (1.0-1.5) | 0.031 | 0.7 (0.5-1.0) | 0.022 |  | 1.8 (1.2-2.6) | 0.003 | 1.3 (0.8-2.3) | 0.322 |
| Physical inactivity (MET < 10) | 1.9 (1.5-2.3) | <0.001 | 1.4 (1.1-1.8) | 0.003 |  | 2.1 (1.4-3.1) | <0.001 | 1.6 (1.0-2.4) | 0.034 |
| **Stressful life events** |  |  |  |  |  |  |  |  |  |
| Family-related events | 2.5 (1.6-4.0) | <0.001 | 1.8 (1.1-3.0) | 0.019 |  | 3.0 (1.4-6.5) | 0.005 | 2.1 (0.9-4.7) | 0.086 |
| Finance related events | 1.3 (0.6-3.2) | 0.517 | 1.3 (0.6-3.2) | 0.523 |  | 1.2 (0.3-4.8) | 0.802 | 1.2 (0.3-4.9) | 0.804 |
| Family member mental disorders | 1.1 (0.5, 2.2) | 0.844 | 1.0 (0.5-2.0) | 0.973 |  | 1.0 (0.2-4.0) | 0.993 | 0.9 (0.2-3.7) | 0.885 |
| **Physical health status** |  |  |  |  |  |  |  |  |  |
| Low BMI | 1.7 (1.4-2.1) | <0.001 | 1.5 (1.2-1.8) | <0.001 |  | 1.8 (1.3-2.6) | 0.001 | 1.8 (1.2-2.5) | 0.002 |
| Major physical illnesses (current) | 2.0 (1.6-2.5) | <0.001 | 1.6 (1.3-2.0) | <0.001 |  | 1.7 (1.1-2.7) | 0.012 | 1.4 (0.9-2.2) | 0.129 |
| Self-rated poor health | 2.4 (1.9-3.0) | <0.001 | 2.1 (1.7-2.7) | <0.001 |  | 2.0 (1.2-3.3) | 0.009 | 1.8 (1.1-3.1) | 0.025 |
| **Mental health status** |  |  |  |  |  |  |  |  |  |
| Depressive disorders | 2.5 (1.3-5.1) | 0.009 | 2.4 (1.2-4.8) | 0.014 |  | 3.7 (0.9-15.0) | 0.067 | 3.4 (0.8-13.8) | 0.090 |
| Anxiety disorders | 2.9 (1.1-7.8) | 0.034 | 3.0 (1.1-8.0) | 0.030 |  | nc |  | nc |  |
| Sleep disorders | 1.6 (1.3-2.0) | <0.001 | 1.4 (1.1-1.7) | 0.006 |  | 1.7 (1.1-2.7) | 0.024 | 1.6 (1.0-2.5) | 0.050 |
| Schizophrenia-spectrum disorders | 8.6 (5.1-14.4) | <0.001 | 9.3 (5.55-15.6) | <0.001 |  | 19.4 (8.5-44.3) | <0.001 | 17.9 (7.7-41.7) | <0.001 |
| Psychiatric disorders (ever) | 7.1 (3.8-13.3) | <0.001 | 7.5 (4.0-14.0) | <0.001 |  | 18.0 (8.3-38.7) | <0.001 | 17.2 (7.9-37.5) | <0.001 |
| Psychiatric disorders (current) | 12.2 (5.8-25.8) | <0.001 | 14.1 (6.6-29.7) | <0.001 |  | 31.1 (13.6-70.9) | <0.001 | 29.3 (12.7-67.7) | <0.001 |
| Unsatisfied with life | 1.6 (1.0-2.7) | 0.036 | 1.7 (1.1-2.8) | 0.029 |  | 1.9 (1.0-3.4) | 0.039 | 1.7 (0.9-3.2) | 0.084 |

Notes: cHR = crude hazard ratio (not adjusted for any covariates). aHR = adjusted HR. nc, model did not converge as the number of individuals from the rural with anxiety disorders was 0. MET: Metabolic Equivalent Task. BMI: body mass index. Prior physical illnesses included diabetes, CHD, stroke or TIA, hypertension, rheumatic heart disease, TB, emphysema/bronchitis, asthma, cirrhosis/chronic hepatitis, peptic ulcer, gallstone/gallbladder disease, kidney disease, fracture, rheumatoid arthritis, neurasthenia, head injury, and cancer.
