# Supplementary material for: Factors associated with suicide risk among Chinese adults: A prospective cohort study of 0.5 million individuals
Source: PLoS Med. 2021 Mar 11;18(3):e1003545. doi: 10.1371/journal.pmed.1003545 (PMC7951865; doi:10.1371/journal.pmed.1003545)
Supplement: S3 Table — (DOCX) [file pmed.1003545.s005.docx]

Supplementary Table 3. Crude hazard ratios for suicide and possible suicide by sociodemographic factors, lifestyle factors, stressful life events, physical and mental health status

|  | Suicide | | Possible Suicide | |
| --- | --- | --- | --- | --- |
|  | cHR (95% CI) | *p* | cHR (95% CI) | *p* |
| **Sociodemographic factors** |  |  |  |  |
| Sex (Male vs Female) | 1.6 (1.3-1.9) | <0.001 | 2.4 (2.0-2.9) | <0.001 |
| Age (10-year band) * | 1.5 (1.4-1.6) | <0.001 | 1.6 (1.5-1.7) | <0.001 |
| Rural residence | 3.1 (2.5-3.8) | <0.001 | 2.5 (2.1-3.1) | <0.001 |
| Low education (< 6years) | 2.7 (2.2-3.3) | <0.001 | 2.1 (1.8-2.6) | <0.001 |
| Low income | 2.0 \|(1.7-2.4) | <0.001 | 1.8 (1.5-2.2) | <0.001 |
| Single | 2.3 (1.8-2.9) | <0.001 | 2.5 (2.0-3.1) | <0.001 |
| Living alone | 3.0 (2.1-4.1) | <0.001 | 2.4 (1.7-3.4) | <0.001 |
| **Lifestyle factors** |  |  |  |  |
| Problem drinking | 1.1 (0.9-1.4) | 0.128 | 2.3 (1.6-3.2) | <0.001 |
| Ever regular smoker | 1.4 (1.2-1.7) | <0.001 | 2.3 (2.0-2.8) | <0.001 |
| Physical inactivity (MET < 10) | 1.7 (1.4-2.0) | <0.001 | 1.6 (1.3-1.9) | <0.001 |
| **Stressful life events** |  |  |  |  |
| Family-related events | 2.5 (1.6-3.7) | <0.001 | 1.8 (1.2-2.8) | 0.009 |
| Finance related events | 1.1 (0.5-2.4) | 0.773 | 0.3 (0.1-1.2) | 0.082 |
| Family member mental disorders | 1.0 (0.6-1.9) | 0.905 | 1.2 (0.7-2.1) | 0.591 |
| **Physical health status** |  |  |  |  |
| Low BMI | 2.0 (1.7-2.3) | <0.001 | 1.6 (1.4-1.9) | <0.001 |
| Major physical illnesses (current) | 1.7 (1.4-2.1) | <0.001 | 1.3 (1.0-1.6) | 0.020 |
| Self-rated poor health | 2.4 (2.0-3.0) | <0.001 | 1.5 (1.2-1.9) | 0.001 |
| **Mental health status** |  |  |  |  |
| Depressive disorders | 3.0 (1.6-5.5) | 0.001 | 0.5 (0.1-2.2) | 0.383 |
| Anxiety disorders | 3.0 (1.1-7.9) | 0.031 | 0.7 (0.1-4.8) | 0.690 |
| Sleep disorders | 1.7 (1.4-2.1) | <0.001 | 1.2 (1.0-1.5) | 0.052 |
| Schizophrenia-spectrum disorders | 10.8 (7.0, 16.7) | <0.001 | 5.7 (3.2-10.1) | <0.001 |
| Psychiatric disorders (ever) | 9.2 (5.7-14.9) | <0.001 | 5.5 (3.0-10.0) | <0.001 |
| Psychiatric disorders (current) | 15.6 (9.0-27.1) | <0.001 | 2.2 (0.6-7.5) | 0.209 |
| Unsatisfied with life | 1.4 (0.9-2.0) | 0.115 | 0.8 (0.5-1.3) | 0.484 |

Notes: cHR, crude hazard ratio (not adjusted for any covariates). MET: Metabolic Equivalent Task. BMI: body mass index. Prior physical illnesses included diabetes, CHD, stroke or TIA, hypertension, rheumatic heart disease, TB, emphysema/bronchitis, asthma, cirrhosis/chronic hepatitis, peptic ulcer, gallstone/gallbladder disease, kidney disease, fracture, rheumatoid arthritis, neurasthenia, head injury, and cancer.
