# Supplementary figures and images for: Factors associated with suicide risk among Chinese adults: A prospective cohort study of 0.5 million individuals
Source: PLoS Med. 2021 Mar 11;18(3):e1003545. doi: 10.1371/journal.pmed.1003545 (PMC7951865; doi:10.1371/journal.pmed.1003545)

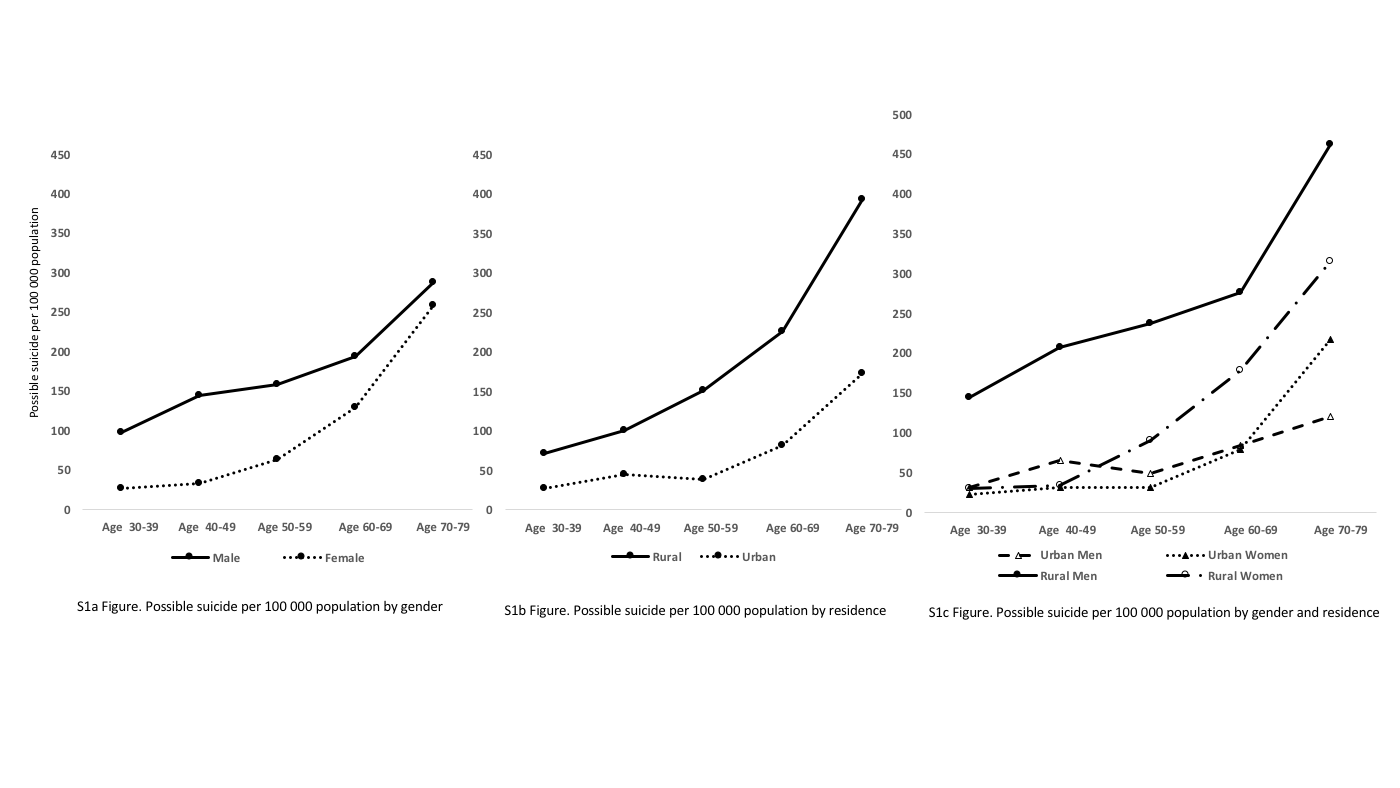

Supplement: S1 Fig — (TIF) [file pmed.1003545.s006.tif]

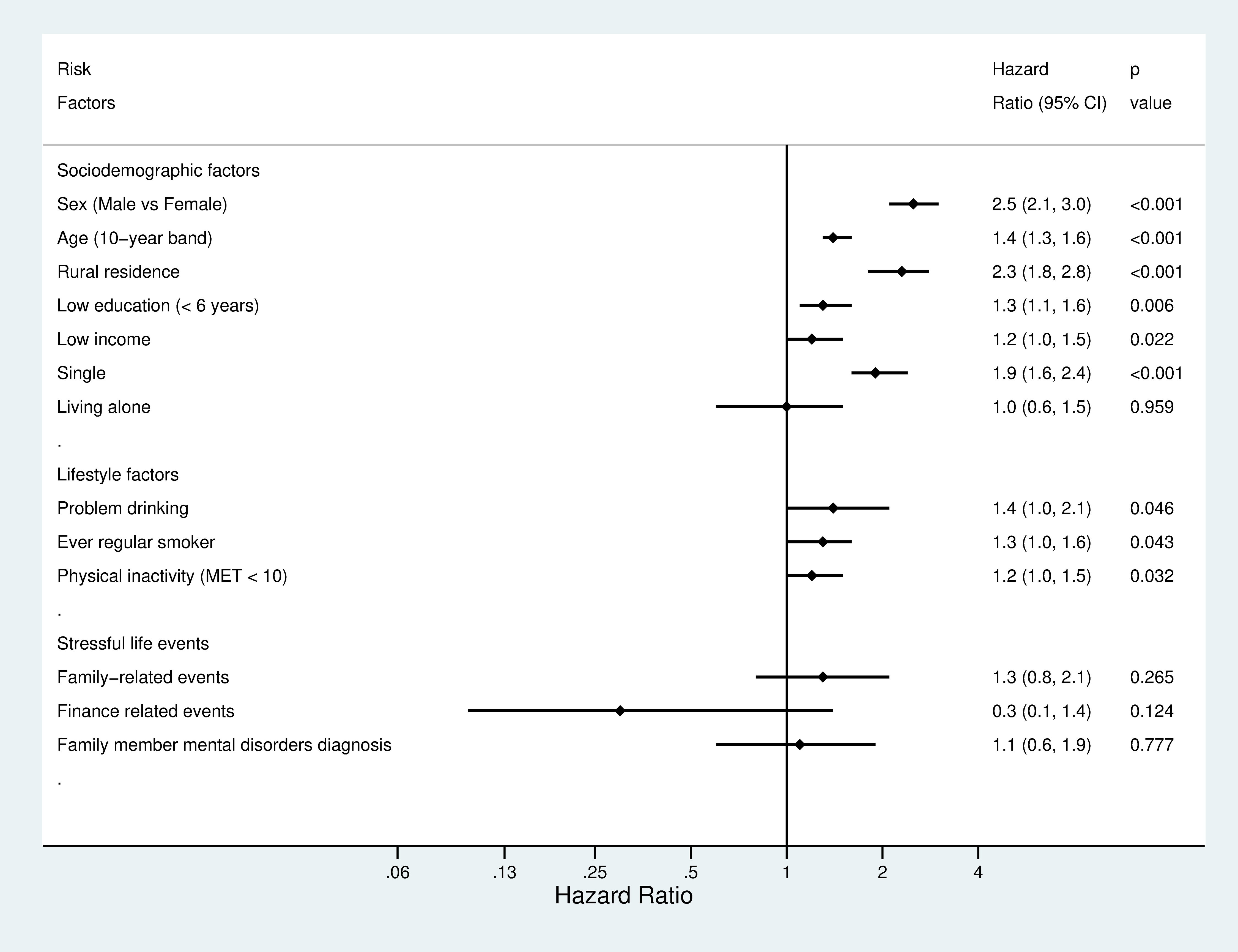

Supplement: S2 Fig — HRs adjusted for sociodemographic factors including sex, age, rural residence, education, income, and single status. HR, hazard ratio; MET, Metabolic Equivalent Task. (TIF) [file pmed.1003545.s007.tif]

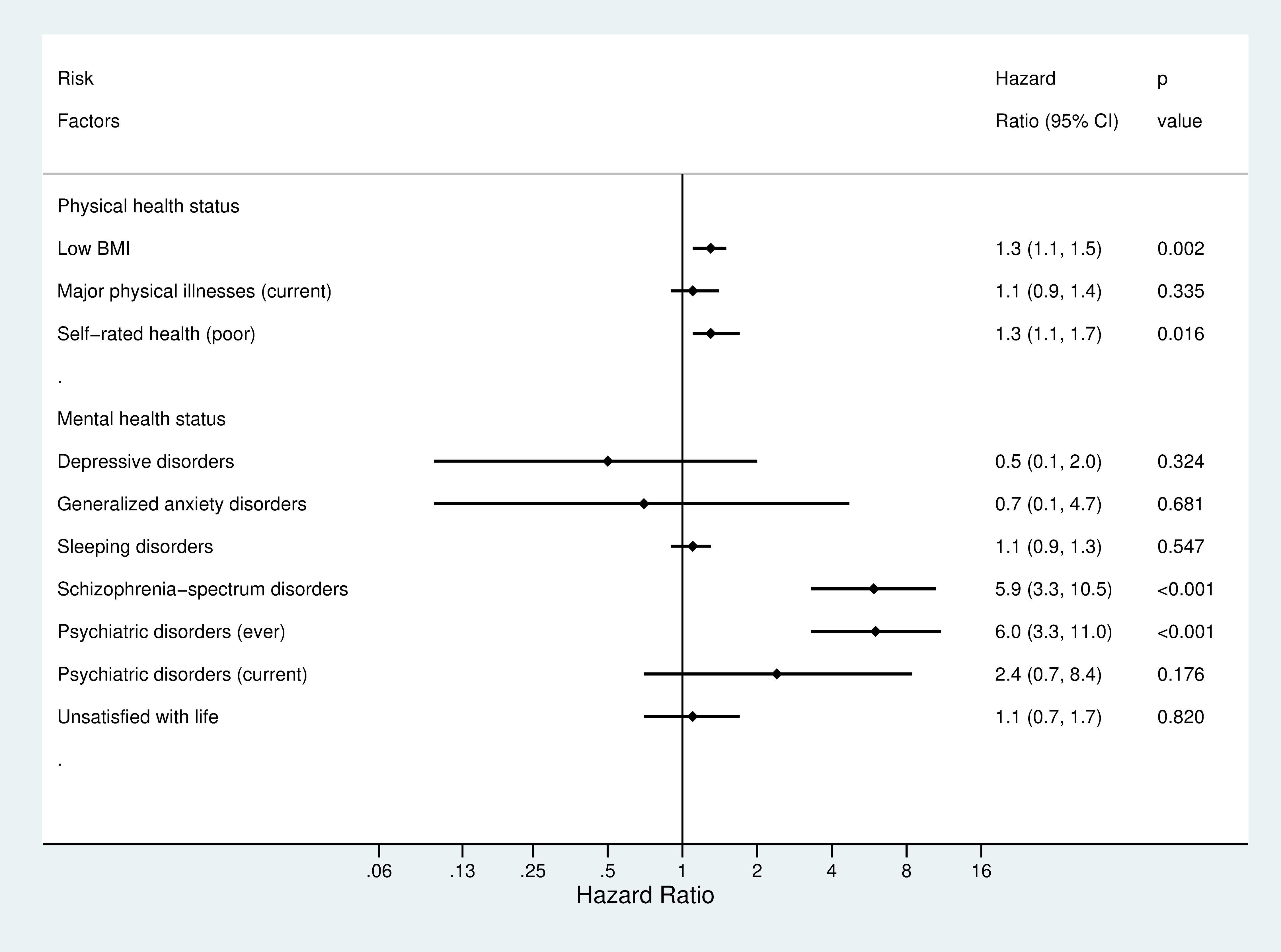

Supplement: S3 Fig — HRs adjusted for sociodemographic factors including sex, age, rural residence, education, income, and single status. HR, hazard ratio; BMI, body mass index. (TIF) [file pmed.1003545.s008.tif]
